# A chromosome-level genome assembly of *Cairina moschata* and comparative genomic analyses

**DOI:** 10.1186/s12864-021-07897-4

**Published:** 2021-07-30

**Authors:** Fan Jiang, Yaoxin Jiang, Wenxuan Wang, Changyi Xiao, Ruiyi Lin, Tanghui Xie, Wing-Kin Sung, Shijun Li, Ivan Jakovlić, Jianhai Chen, Xiaoyong Du

**Affiliations:** 1 Hubei Key Laboratory of Agricultural Bioinformatics, College of Informatics, Huazhong Agricultural University, 430070 Wuhan, People’s Republic of China; 2 Key Lab of Agricultural Animal Genetics, Breeding and Reproduction, Ministry of Education, College of Animal Science and Technology, Huazhong Agricultural University, 430070 Wuhan, People’s Republic of China; 3 College of Animal Science, Fujian Agriculture and Forestry University, 350002 Fuzhou, China; 4 Department of Computer Science, National University of Singapore, Singapore, Singapore; 5 Joint International Research Laboratory of Modern Agricultural Technology, Ministry of Education, Jilin Agricultural University, 130118 Changchun, China; 6 State Key Laboratory of Grassland Agro-Ecosystem, Institute of Innovation Ecology, Lanzhou University, 730000 Lanzhou, China; 7 Institutes for Systems Genetics, Frontiers Science Center for Disease-related Molecular Network, West China Hospital, Sichuan University, Sichuan Chengdu, China

## Abstract

**Background:**

The Muscovy duck (*Cairina moschata*) is an economically important duck species, with favourable growth and carcass composition parameters in comparison to other ducks. However, limited genomic resources for Muscovy duck hinder our understanding of its evolution and genetic diversity.

**Results:**

We combined linked-reads sequencing technology and reference-guided methods for *de novo* genome assembly. The final draft assembly was 1.12 Gbp with 29 autosomes, one sex chromosome and 4,583 unlocalized scaffolds with an N50 size of 77.35 Mb. Based on universal single-copy orthologues (BUSCO), the draft genome assembly completeness was estimated to be 93.30 %. Genome annotation identified 15,580 genes, with 15,537 (99.72 %) genes annotated in public databases. We conducted comparative genomic analyses and found that species-specific and rapidly expanding gene families (compared to other birds) in Muscovy duck are mainly involved in Calcium signaling, Adrenergic signaling in cardiomyocytes, and GnRH signaling pathways. In comparison to the common domestic duck (*Anas platyrhynchos*), we identified 104 genes exhibiting strong signals of adaptive evolution (Ka/Ks > 1). Most of these genes were associated with immune defence pathways (e.g. *IFNAR1* and *TLR5*). This is indicative of the existence of differences in the immune responses between the two species. Additionally, we combined divergence and polymorphism data to demonstrate the “faster-Z effect” of chromosome evolution.

**Conclusions:**

The chromosome-level genome assembly of Muscovy duck and comparative genomic analyses provide valuable resources for future molecular ecology studies, as well as the evolutionary arms race between the host and influenza viruses.

**Supplementary Information:**

The online version contains supplementary material available at 10.1186/s12864-021-07897-4.

## Background

The Muscovy duck (*Cairina moschata*), one of the largest wood ducks, is more land-oriented than other ducks [1]. Domestic Muscovy duck has become one of the most economically important poultry species in the world due to its unique taste, high breast meat production and low calories. Eleven breeds of Muscovy duck in Latin America, the Caribbean, Europe, Asia Pacific and Africa are registered in the Domestic Animal Diversity Information System [2]. Muscovy duck was domesticated in Central or South America, spread around the world by European colonists, and eventually introduced to China [3, 4]. The breeding history of Muscovy duck in China has a reliable record of more than 250 years [3]. Except for Muscovy duck, almost all varieties of domesticated ducks are descended from mallard (*Anas platyrhynchos*) [5]. The genome of the common domestic duck has been characterized and published [6]. The Muscovy duck genome (assembly version: CaiMos1.0) was sequenced in 2019 using second-generation sequencing data and a reference-guided method, but it lacks accuracy evaluation, and it remains unpublished (GenBank acc. no. GCA_009194515.1).

The modern technology of next-generation sequencing can generate billions of short-read fragments at a relatively low price with high accuracy [7]. It is difficult to obtain long and continuous scaffolds using short reads, owing to repetitive or heterozygous structures, which makes *de novo* assembly challenging [8]. Although the use of PacBio long-read sequencing, Bionano optical mapping, and Hi-C scaffolding can provide highly contiguous genome assemblies, those methods substantially increase the cost of sequencing projects. Linked-reads sequencing (10x Genomics) [9] has recently been shown to generate a high-quality, cost-effective de novo assembly in a non-model mammal [10]. In addition, combining 10x genomics and a reference-guided method can achieve chromosome-scale assembly from a previous study [11] without the need for mate-pair reads with different insert sizes, or physical and genetic maps [12].

Here, we combined linked-reads sequencing (10x Genomics) and reference-guided approaches as a cost-effective strategy to enhance the conventional short-read and long-read-based methods, to obtain a draft assembly of the Muscovy duck genome. Comparative genomics analyses of the Muscovy duck, including orthology, species-specific, rapid expansion and identification of positively selected genes could deepen our understanding of the evolutionary relationships between different species of birds at the molecular level. The comparison of the evolution of sex chromosomes and autosomes could shed light on the fundamental evolutionary forces. Our draft assembly is available for public use, and genome analysis can assist future studies of evolution and ecology in birds.

## Results and discussion

### Genome assembly

A total of ~ 128-fold (128 Gbp data) read coverage was obtained with paired-end 150 bp reads using 10x Chromium technology in this project. Using the Supernova [9] assembler, we produced a draft Muscovy duck genome of 1.12 Gbp. This is lower than the predicted 1.32 Gbp, based on kmer analysis (Additional file 1: Fig. S1). The assembly comprised 15,925 scaffolds > 1 kb with contig N50 of 219.51 kb and scaffold N50 of 2.27 Mb. The mean input DNA molecule length (~ 16.65 Kb) in Supernova assembler statistics output (Additional file 2: Table S1) was lower than the official recommendation (50–100 kb), which may greatly affect the scaffold N50 length. To further assemble these into a contiguous draft, we reused the 10x reads for kmer mapping against the Supernova assembler. Our results showed that ARKS software [13] further improved the connectivity of the Supernova genome (scaffold N50 = 5.22 Mb).

The reference-based scaffolder Chromosomer [12] was used to align previous scaffolds to the closely related *A. platyrhynchos* genome to construct a chromosome-level assembly. After reference-guided scaffolding, approximately 99.08 % of genome sequences assembled by ARKS were aligned to the *A. platyrhynchos* genome; 93.98 % of the sequences were anchored to 29 autosomes and sex chromosomes. GapCloser [14] was then used to fill the gaps in the pseudo-chromosomes, resulting in 13,924 gaps completed.

Finally, we assembled a chromosome-level Muscovy duck genome of 1.12 Gbp, including 4,613 scaffolds > 1 kb with scaffold N50 of 77.35 Mb. The total genome size is comparable to that of the *A. platyrhynchos* (~ 1.13 Gbp). We evaluated the completeness of the draft genome assembly by calculating coverage for a set of Single-Copy Orthologous genes in Aves using BUSCO [15]. The result showed that the genome coverage rate is 93.30 %, slightly higher than *A. platyrhynchos*’s genome completeness (89.10 %) (Table 1).

**Table 1:** Assembly statistics for the *Cairina moschata* genome sequence reported in this study and comparison to the previously sequenced *Anas platyrhynchos* genome

Assembly	*Cairina moschata*	*Anas platyrhynchos*
Contig N50	326,005	2,706,497
Scaffold N50	77,345,420	76,129,154
Scaffold N90	9,745,227	10,039,220
Number of scaffolds ( > = 1Kb)	4,613	2,150
Number of long scaffolds ( > = 1 Mb)	39	30
Largest scaffold (bp)	194,810,853	202,842,836
Total scaffold size (bp)	1,118,556,028	1,126,176,092
GC/N (%)	41.02/1.34	41.53/0.26
BUSCO genome completeness	C:93.3 %,F:2.4 %,M:4.3 %	C:89.1 %,F:3.1 %,M:7.8 %

Note: *BUSCO* Benchmarking Universal Single-Copy Orthologs, *C* Complete BUSCOs, *F* Fragmented BUSCOs, *M *Missing BUSCO

*Anas platyrhynchos* genome (IASCAAS_PekingDuck_PBH1.5, 2018)

### Gene prediction and annotation

We predicted 15,580 protein-coding genes by integrating *ab initio*, homology- and transcript-based methods. The number and length of genes and exons predicted by each approach are listed in Additional file 3: Table S2. Subsequently, we aligned the protein sequences to the BUSCO database to evaluate the annotation quality, and found 90.8 % single copy BUSCO genes (Additional file 4: Table S3), suggesting a high degree of completeness in the predicted genes. For repetitive elements, the result showed that the level of repeats in the Muscovy duck genome (9.19 %, Additional file 5: Table S4) is similar to that of the chicken genome (9.45 %) and higher than that of the *A. platyrhynchos* genome (5.85 %) [6]. In birds, transposable elements (TEs) usually account for 4–10 % of the total genome size [16]. Finally, 99.72 % (15,537) of the predicted genes were functionally annotated using public databases (Additional file 6: Fig. S2). Taken together, these analyses suggested a satisfying level of completeness and accuracy of genome annotation.

### Orthology and evolution

**Fig. 1:**
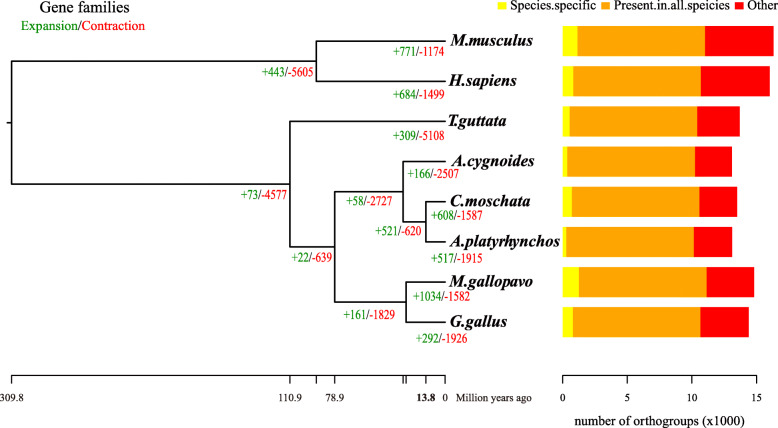
Phylogeny with split times and gene family expansion/contraction in the Muscovy duck and a set of related species. The phylogeny was estimated using a maximum likelihood analysis of a concatenation of 500 single copy orthologous protein sequences over 100 bootstrap replicates. *Mus musculus* and *Homo sapiens* were used as outgroups. Predicted species split times are plotted at each node. The numbers of expanded (green) and contracted (red) gene families are shown on branches. Horizontal bar plots (right) indicate the number of orthogroups that are species-specific (yellow), present in all 8 species (orange), or present in more than one but less than all species in the analysis (red)

The OrthoFinder [17] analysis identified 22,701 orthogroups (gene family clusters), of which 16,783 comprised two or more species (Additional file 7: Table S5). Of the 13,500 orthogroups including one or more Muscovy duck sequences, 9,878 orthogroups (43.51 %) were present in all species (Fig. 1). Among these, 7,182 consisted entirely of single-copy genes with a one-to-one relationship in different genomes (Additional file 7: Table S5). Compared to other species, we found 762 Muscovy duck-specific genes, classified into 709 gene families (Additional file 8: Table S6). To further elucidate the biological relevance of these species-specific genes, we assigned Gene Ontology (GO) terms using WEGO 2.0 [18]. GO analysis indicated that most of these genes were classified into the molecular function category, including functional subcategories such as ion binding, protein binding, heterocyclic compound binding, organic cyclic compound binding, etc. (Additional file 9: Fig. S3). In addition, we also performed a Kyoto Encyclopedia of Genes and Genomes (KEGG) analysis [19] using these genes and found 24 mainly enriched pathways, including Calcium signaling pathway, Vascular smooth muscle contraction, Gap junction, Focal adhesion, Adherens junction, etc. (Additional file 10: Table S7). Furthermore, we also found that Muscovy-specific genes (*GABRG3, MDGA1* and *Shank3*) were associated with the development of the brain and nervous system. Indeed, compared to common domestic duck, Muscovy duck had higher virus titers developed in vital organs, particularly in the brain [20]. It is an interesting question to investigate Muscovy-*A. platyrhynchos* differences in brain morphology, worthy of further study.

The divergence time tree using 500 (6.96 %) single-copy genes dated the common ancestor of Muscovy and *A. platyrhynchos* to about 13.8 million years ago (MYA) (Fig. 1), which is within the range proposed by previous studies (9.0–17.9 MYA) [21–23]. Based on the results of gene cluster analysis in the previous step, we performed a computational analysis of gene family size to understand gene family expansion and contraction between the Muscovy duck and the other seven species included in the dataset for comparative analyses. We found that ~ 608 gene families had undergone expansion (517 in *A. platyrhynchos*) and ~ 1,587 gene families had contracted (1,915 in *A. platyrhynchos*) (Fig. 1). A total of 134 significantly expanded gene families and 529 contracted gene families were found in the Muscovy duck (*P* < 0.05) (Additional file 11: Table S8). KEGG analysis of 479 genes from these expanded gene families revealed that they were mainly classified as ABC transporters, Calcium signaling pathway, Adrenergic signaling in cardiomyocytes, and GnRH signaling pathway (Table 2). Intriguingly, some of these pathways overlapped with pathways that species-specific Muscovy duck genes were involved in, such as the Calcium signaling pathway, Adrenergic signaling in cardiomyocytes, and GnRH signaling pathway (Table 2 and Additional file 10: Table S7). In addition, we found that the *A. platyrhynchos* genome had 1060 genes corresponding to 529 gene families, while the Muscovy duck genome only existed 39 genes (Additional file 11: Table S8). These significantly contracted genes of Muscovy duck were involved in 4 pathways: Necroptosis, Histidine metabolism, β-Alanine metabolism, and Ascorbate and Aldarate metabolism (Table 2). We also found 39 genes were mainly annotated in olfactory receptor (Additional file 12: Table S9). Gene family expansion and contraction is often reflective of phenotypic adaptation during the evolutionary trajectory of species [24].

**Table 2:** Functional annotation of the significant expanded and contracted gene families in Muscovy duck

Gene families	KEGG terms	Genes number	*P* value
Expanded gene families	ABC transporters	12	1.97E-10
	Calcium signaling pathway	18	3.32E-08
	Adrenergic signaling in cardiomyocytes	14	2.31E-07
	GnRH signaling pathway	12	3.94E-07
	Arginine and proline metabolism	7	1.29E-05
	Adherens junction	9	1.52E-05
	Pentose and glucuronate interconversions	5	4.86E-05
	Neuroactive ligand-receptor interaction	17	2.81E-04
	Biosynthesis of amino acids	7	3.03E-04
	Tight junction	11	3.46E-04
	Cell adhesion molecules (CAMs)	9	4.00E-04
	Fructose and mannose metabolism	5	4.26E-04
	Vascular smooth muscle contraction	9	5.44E-04
	Regulation of actin cytoskeleton	11	9.36E-04
	Ubiquitin mediated proteolysis	9	9.59E-04
	Focal adhesion	11	1.54E-03
	Melanogenesis	7	2.98E-03
	Retinol metabolism	4	3.39E-03
	Fatty acid biosynthesis	3	4.49E-03
	Protein processing in endoplasmic reticulum	8	7.15E-03
	ECM-receptor interaction	6	7.38E-03
	Cardiac muscle contraction	5	9.44E-03
	Oocyte meiosis	6	1.21E-02
	Intestinal immune network for IgA production	3	2.06E-02
	Mitophagy - animal	4	2.17E-02
	Propanoate metabolism	3	2.43E-02
	Pyruvate metabolism	3	2.63E-02
	Herpes simplex virus 1 infection	6	3.79E-02
	Primary bile acid biosynthesis	2	4.05E-02
Contracted gene families	Necroptosis	17	5.71E-05
	Histidine metabolism	5	5.31E-03
	β-Alanine metabolism	5	1.81E-02
	Ascorbate and aldarate metabolism	3	2.02E-02

Since Ka/Ks values > 1 are considered to indicate the directional (Darwinian) evolution [25], we focused on 104 genes with Ka/Ks ratios greater than 1.0 in the Muscovy duck genome as further evidence of adaptive evolution (Additional file 13: Table S10). KEGG annotation results showed that rapidly evolving genes were enriched in the Toll-like receptor, Cytokine-cytokine receptor interaction, Necroptosis and Influenza A signaling pathways (Additional file 14: Table S11). We found that two interferon genes (*IFNAR1* and *IFNAR2*) were simultaneously involved in these four pathways and that the tumor necrosis factor (TNF) gene and a *TLR5* gene were independently involved in the Cytokine signaling pathway and the Toll-like receptor pathway, respectively (Additional file 15: Table S12). Gu et al. [26] also found that *IFNAR1* and *TLR5* exhibited Muscovy-specific SNPs. Signatures of positive selection and species-specific SNPs both indicated that these two Muscovy duck genes underwent adaptive evolution. All of these pathways play important roles in the innate immune response mechanisms [27, 28]. For example, the expression of Toll-like receptor (TLR) genes was mostly up-regulated in the brain after the duck plague virus infection (Anatid alphaherpesvirus 1), and expression levels of cytokine-related (IFNA, TNF) and TLR genes were significantly increased in the lungs after the avian influenza virus infection [6, 29]. We identified five TLRs (*TLR3, TLR4, TLR5, TLR7, TLR15*) in the Muscovy duck genome through annotation to *A. platyrhynchos* genome. These genes with Ka/Ks value less than 1 except for the *TLR5*, which indicates that most Toll-like-related genes experienced purifying selection in the Muscovy duck genome. Furthermore, a relatively high number of directional evolution genes related to immune response indicate that Muscovy duck might have a stronger immune system than *A. platyrhynchos*. However, contrary to this prediction, the Muscovy duck is more susceptible to avian influenza virus H5N1 in terms of disease development and mortality than *A. platyrhynchos* [20, 30]. Arguably, this one case-study may not be reflective of the overall immune system responses in these two species. Further studies of these genes involved in the innate immune response pathways may provide insights into the viral defense mechanisms in Muscovy ducks.

### Syntenic relationship with the A. platyrhynchos genome

**Fig. 2:**
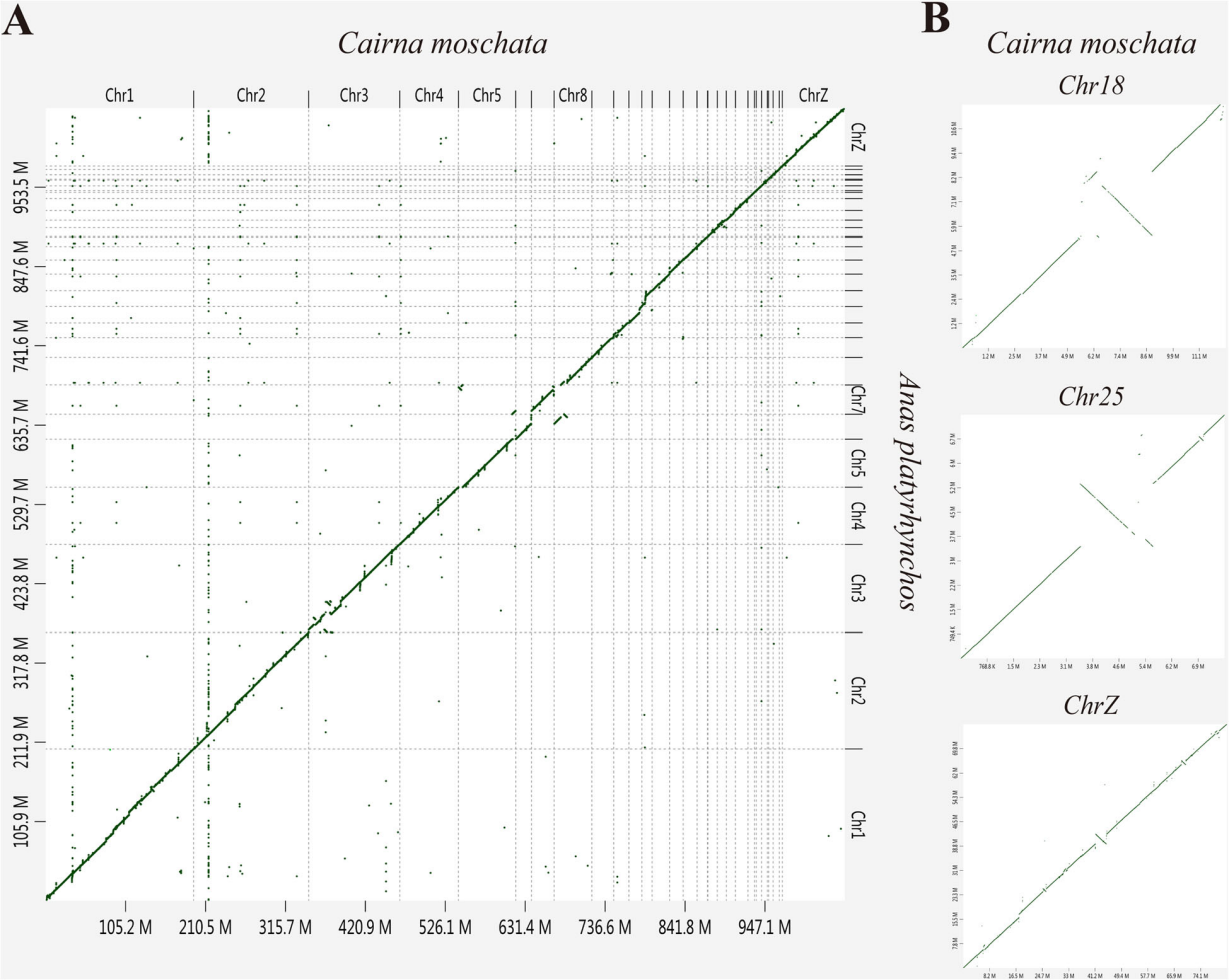
D-GENIES dot plot showing the syntenic relationships between Muscovy duck and *A. platyrhynchos*. **a** All autosomes and sex chromosomes. **b** Focused views of the chromosomes 18, 25, and Z. The X-axis represents the Muscovy duck chromosome and Y-axis represents the *A. platyrhynchos* chromosome. A diagonal straight line indicates synteny among the genomes

To assess the syntenic relationship between species, we aligned the draft Muscovy duck genome assemblies to the *A. platyrhynchos* genome, which is currently the closest species with available chromosome-level assembly. The dot plot showed that reference-based pseudochromosomes exhibited a highly collinear relationship with the *A. platyrhynchos* chromosomes (Fig. 2), which indicated a high quality of our genome assembly. Several large-scale rearrangements were observed between the two genomes, including interspecific inversions at the Chr18: 6.49–8.89 Mb, Chr25: 3.45–5.59 Mb, and ChrZ (the sex chromosome): 41.33–44.69 Mb. To evaluate the reliability of these inversions, we independently checked them using structural variation detection software. Several small inverted regions were detected by BreakDancer [31] (Additional file 16: Table S13) within the largest inversion detected by SyRI [32] (Fig. 1 and Additional file 17: Table S14). SyRI detected an inversion at the chromosome level, while Breakdancer detected an inversion in a short read sequence, which indirectly confirmed the SyRI results. The ChrZ inversion with 3.36 Mb was the largest one, and the number of inversion on the Z chromosome was significantly greater than in all autosomes when chromosome size was accounted for (Additional file 18: Fig. S4). This is probably indicative of the comparatively fast evolution of the Z chromosome, known as the “faster-Z effect” [33, 34].

### Faster-Z effect and inversion polymorphisms

Several studies have found a greater divergence in coding sequences on sex chromosomes (ZZ/ZW) than on the autosomes in birds [1–4]. Nonsynonymous to synonymous substitutions ratio (dN/dS = ω) can provide an insight into the strength of purifying and directional selection [5, 6]. We obtained 7,516 Muscovy - *A. platyrhynchos* 1:1 orthologs, encompassing 12.66 Mb, and found significantly higher mean ω values in genes linked to the Z chromosome compared to autosomes (*P* = 2.20E-07, based on two-sided Wilcoxon tests; Table 3). In agreement with previous findings [35, 36], this was driven by an increase in dN (*P* = 1.55E-06), rather than a decrease in dS on the Z chromosome. Two alternative explanations are codon bias differences between sex chromosomes and autosomes [37], and weak selection at synonymous sites [38]. The Faster-Z effect can be explained by the fact that the effective population size of Z chromosomes is less than that of autosomes, which in turn will lead to increased genetic drift [35, 39]. We performed a re-sequencing data analysis and identified 6,186,165 polymorphic loci (including 295,278 loci on the Z chromosome) from Muscovy duck populations and 23,124,940 polymorphic loci (including 1,089,826 on the Z chromosome) from mallard populations. Muscovy and mallard populations clustered as two genetically distinct groups in a phylogenetic tree (Additional file 19: Fig. S5). The F_ST_ values between Muscovy and mallard ducks were calculated for Z chromosomes and autosomes. This analysis revealed that divergence between the two species was higher for the Z chromosome (F_ST_ = 0.7838) than for autosomes (0.6286). Synonymous sites can be used to approximate the level of neutral polymorphism to assess the effective population size (Ne) [40]. The synonymous nucleotide diversity (πs) values of Z-linked genes in the Muscovy duck and mallard were lower than those of autosomal genes (Table 4). This reflects a lower Ne on the Z chromosome than autosomes in both species, which in turn is expected to result in decreased strength of purifying selection on the Z chromosome than autosomes, as purifying selection is less powerful at lower effective population sizes [41]. The average πsZ/πsA estimates for Muscovy duck (0.730) and mallard (0.7006) were slightly lower than the neutral expectation of 0.75. The loss of diversity could be explained by increased genetic drift on the Z chromosome [39].

**Table 3:** Non-synonymous substitution rates (dN) and synonymous substitution rates (dS), and dN/dS values of Z-linked and Autosomal genes

	Number of Loci	Mb	dN	dS	dN/dS
Z-linked	466	0.77	0.025[0.021; 0.029]	0.078[0.067; 0.088]	0.293[0.268; 0.318]
Autosomal	7050	11.89	0.019[0.018; 0.020]	0.078[0.073; 0.082]	0.237[0.230; 0.243]

Note: Intervals represent 95 % confidence intervals obtained by bootstrapping with 1000 replicates

**Table 4:** Nucleotide diversity (π), non-synonymous (πn) and synonymous (πs) nucleotide diversity values from Z-linked and Autosomal Genes

	Muscovy duck	Mallard duck
Z-linked		
π	4.028e-01 [4.024e-01; 4.033e-01]	3.984e-01 [3.980e-01; 3.989e-01]
πn	1.653e-04 [1.284e-04; 2.022e-04]	1.217e-04 [9.237e-05; 1.511e-04]
πs	2.098e-04 [1.681e-04; 2.515e-04]	2.020e-04 [1.616e-04; 2.425e-04]
Autosomal		
π	4.015e-01 [4.013e-01; 4.016e-01]	3.235e-01 [3.234e-01; 3.236e-01]
πn	1.543e-04 [1.469e-04; 1.618e-04]	1.112e-04 [1.057e-04; 1.168e-04]
πs	2.874e-04 [2.784e-04; 2.965e-04]	2.884e-04 [2.794e-04; 2.973e-04]
πsZ/πsA	0.730	0.701

Note: Intervals represent 95% confidence intervals obtained by bootstrapping with 1000 replicates

**Fig. 3:**
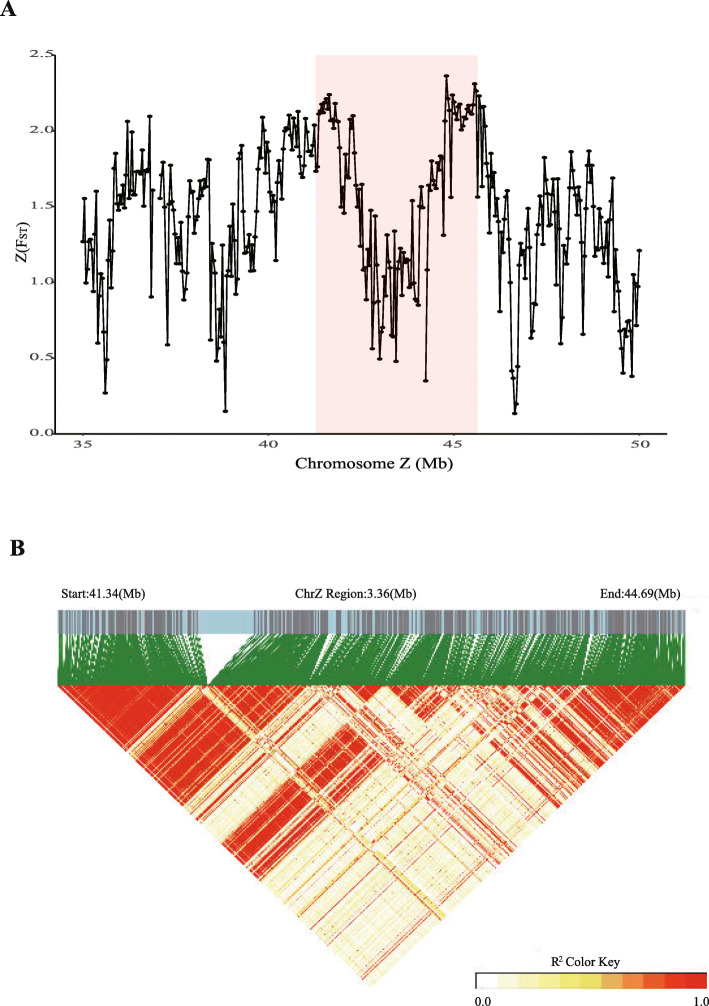
ChrZ inversion polymorphisms. **a** Genome-wide screen for genetic differentiation in the 3.36 Mb inverted region on the Z chromosome between Muscovy duck and mallard using normalized F_ST_ values (ZF_ST_) calculated in 40 kb windows. **b**. LD value in the 3.36 Mb inversion region on the Z chromosome inferred on a mallard population (6 samples)

We then detected Z chromosome inversion polymorphisms and found that the F_ST_ values around the two breakpoints of the largest inversion on the Z chromosome were significantly higher than in adjacent genomic regions (Fig. 3). Similar phenomena have been observed before [42], and they are believed to be a consequence of the fact that inversion inhibits chromosomal recombination, which in turn results in greater genetic differentiation in the vicinity of inverted segments [43, 44]. Similarly, we also observed high LD near the inversion breakpoints as a result of almost completely inhibited recombination in these two regions (Fig. 3). These are in agreement with the hypothesis that inversion polymorphisms can propel the sex evolution as a result of suppressed recombination [45, 46].

## Conclusions

Using linked-reads sequencing data and RNA-seq data we have generated a high-quality draft genome assembly and annotation of the Muscovy duck genome. Synteny analysis showed that reference-based pseudochromosomes exhibited a highly collinear relationship with corresponding common domestic duck chromosomes. Comparative genome analysis of the Muscovy duck and common duck genomes, including orthology, species-specific, rapid expansion and positively selected analyses could deepen our understanding of the evolutionary relationship between these two closely related species at the molecular level. In addition, we found mixed evidence of rapid divergence on the Z chromosome relative to autosomes using divergence and polymorphism data. Strikingly, inversions were enriched on the Z chromosome compared to autosomes, suggesting that inversion polymorphisms propelled the evolution of sex chromosomes. In conclusion, these results deepen our understanding of the evolution and ecology of Muscovy duck.

## Methods

### Samples and sequencing

 A male Muscovy duck was collected in Wuhan, China (Animal handling and experiments were approved by the Scientific Ethic Committee of Huazhong Agricultural University (Permission number: HZAUCA-2016-058)). The liver sample was stored at -80 °C. All experiments and methods were performed in accordance with the ARRIVE guidelines (Animal Research: Reporting of In Vivo Experiments) [47]. We declared that all methods were carried out in accordance with the relevant guidelines and regulations.

The Chromium™ Genome Protocol was used to generate a high molecular weight (HMW) genomic DNA from fresh liver tissues. Sample indexing and partition barcoded libraries were conducted using the Chromium Genome Reagent Kit (10x Genomics) [9] (Novogene Company, Beijing, China) following the manufacturer’s protocol. DNA sequencing on the Illumina NovaSeq platform generated paired-end reads. After removing poly-N and low-quality sequences, 128 Gbp clean reads (~ 128x) with a length of 150 bp were generated, and used for the subsequent genome assembly. Furthermore, the heart, liver, spleen, lung, kidney, brain, chest muscles, lymph, oral mucosa, stomach, small intestine, large intestine and hair follicle tissues were collected from the same duck specimen and used for RNA extraction. The RNA extracted from each of these tissues was mixed (1 µg from each tissue), and used as input material for the library construction workflow, which included the isolation of polyadenylated RNA molecules using poly-T oligo-attached magnetic beads, enzymatic RNA fragmentation, cDNA synthesis, ligation of bar-coded adapters and PCR amplification. Then clustering of the index-coded samples was performed on a cBot Cluster Generation System using TruSeq PE Cluster Kit v3-cBot-HS (Illumina) and sequenced using an Illumina Novaseq platform. After removing reads containing adapter sequences, poly-N, and low-quality reads from raw data, we obtained ~ 20.8 Gbp clean reads with a length of 150 bp to assist genome annotation.

### Genome assembly and reference-guided reconstruction

Approximately 20.8 Gbp clean reads were obtained and then the 16 bp 10x Genomics barcode was trimmed using Long Ranger v2.1 basic. Jellyfish v2.3.0 [48] was used to count the frequency of kmer size of 21. The histogram of the kmer counting distribution was processed in GenomeScope (v-2.0) (kmer_max = 10,000) [49] to estimate the genome size, the abundance of repetitive elements, and heterozygosity.

The original 10x Genomics linked-reads were used as input for the Supernova (v-2.1.1) [9] assembler for *de novo* genome assembly with the maximum reads (-maxreads) parameter set at 858 million input reads with optimal raw coverage of 85-fold, greater than the 56-fold suggested in the Supernova protocol (Additional file 2: Table S1). For further assembly into contiguous drafts, we used ARKS v-1.0.4 (parameters: m = 20–20,000, a = 0.9) [13], which again utilized the original 10x reads for kmer mapping against the Supernova assembly. The companion LINKS [50] program applied a scaffold graph generated by ARKS to create a longer assembly. To remove the artifacts produced by the above step, we removed scaffolds composed entirely of N and stretches of Ns at the beginning and end of scaffolds.

Chromosomer (v-0.1.3) [12] then was used to construct large pseudomolecules corresponding to the chromosomes from the assembled contigs or scaffolds by using *A. platyrhynchos* (IASCAAS_PekingDuck_PBH1.5) as the reference genome. For the assembly process, the scaffold sequences were aligned to the genome of *A. platyrhynchos* genome using BLASTN v 2.6.0 (-outfmt 6 and -evalue 1E-10) [51]. Chromosomer (fragmentmap -r 1.01) software utilizes the results of alignments to connect the mapping fragments with a gap length setting of 100 and anchor them to the reference genome chromosomes. The redundant scaffolds (< 2 Mb) removed by CD-HIT (-c 0.99) [52], unlocalized and unplaced scaffolds were also collected to produce the final assemblies.

Finally, GapCloser (v-1.12) (-l 155 -p 31) [14] was used to fill the gaps left in the assembly process. At each step of the assembly, a Benchmarking Universal Single-Copy Orthologs (BUSCO v-4.0.6) [15] analysis was applied to evaluate the completeness of the gene set in our draft genome with the library “aves_odb10”.

### Gene prediction and annotation

We employed a combined approach utilizing *ab initio*, homology- and transcript-based strategies to predict protein-coding genes. GeneMark-ES [53] and Augustus (v-3.3) [54] were used for *ab initio* gene prediction. GeneMark-ES is an unsupervised training algorithm that identifies protein-coding genes in eukaryotic genomes. Augustus (--species = chicken) was trained to apply homologous protein sequences from the closely related species and our RNA-Seq data to improve the reliability of the *de novo* prediction. Based on the homology method, exonerate (v-2.2.0) [55] (--model protein2geneome --percent 50) was used to align the annotated gene sets from four closely related species, *A. platyrhynchos, Gallus gallus, Meleagris gallopavo* and *Taeniopygia guttata*, to our draft genome. Based on the transcript prediction, Hisat2 (v-2.1.0) [56] was used to align the transcriptome data from 13 tissues to the draft Muscovy duck genome, and Stringtie (v-2.1.3b) [57] was used to predict the gene information. To improve the accuracy of transcription prediction, we also employed the PASA (v-2.4.1) annotation [58] to reconstruct the transcripts, and then TransDecoder (v-5.5.0) (http://transdecoder.sf.net) was utilized to identify potential protein-coding regions. We used EvidenceModeler [59] to integrate the gene structures obtained from the above three methods and filter out genes that lacked homolog identification or RNA-Seq data support. Finally, we aligned the predicted gene structures to the BUSCO (-m prot) databases to evaluate the obtained gene set.

To identify repeat elements in our draft genome we used a combined approach of *de novo* and homology-based prediction. RepeatModeler (v-1.0.11) (http://www.repeatmasker.org/RepeatModeler/) was used to construct the *de novo* self-specificity repeat library, and RepeatMasker (v-4.1.0) [60] was applied to produce a homolog-based repeat library with default parameters.

For gene function annotation, we used the gene sequences to align NCBI non-redundant protein (NR), Nucleotide Sequence (NT) [61], SwissProt [62] with E-value cutoffs of 1E-5. Best-hit BLAST results were then used to determine gene functions. Furthermore, motifs and domains in the predicted gene sequences were annotated using InterProScan (v-5.45) [63] relying on publicly available databases: Gene3D, PRINTS, Pfam, CDD, SMART, and MobiDBLite. PROSITE and Gene Ontology (GO) terms for each gene were extracted from the corresponding InterProcan entries.

### Orthology and evolution

Protein sequences from the entire genome data of 5 species of birds (*Taeniopygia guttata, Anser cygnoides, Anas platyrhynchos, Meleagris gallopavo, Gallus gallus*) and 2 species of mammals (*Mus musculus, Homo sapiens*) were downloaded from the NCBI’s public database (Additional file 20: Table S15) and used for comparative analyses and gene clustering analysis. To produce a single transcript for each protein set, we filtered redundant alternative splicing events. We then identified final orthologs, in-paralogs and co-orthologs for all protein-coding genes using OrthoFinder (v-2.3.12) [17] with default parameters. To reconstruct the phylogenetic relationships among these species, 500 single-copy orthologous protein sequences were selected to perform multiple alignments using MUSCLE (v-3.8.31) [64]. We extracted conserved blocks using Gblocks (v-0.91b) [65] and concatenated them into 8 supergenes. The JTT model in PhyML-3.3 [66] was used to construct a maximum likelihood phylogenetic tree (100 bootstrap replicates). In addition, the MCMCtree program (-clock 2 -alpha 0.5 -model 3, PAML 4.8 package) [67–69] was used to combine the known time-calibration data in TIMETREE (http://www.timetree.org/) to estimate divergence times among species. Based on the results of OrthoFinder and divergence time, we applied CAFE v4.1 [70] with a p-value of 0.05 to identify gene families that underwent expansion and contraction in the Muscovy duck genome compared with the other species. Then, we used the KOBAS [71] program to infer functional information about expanded or contracted gene families. Protein alignments of single-copy Muscovy - *A. platyrhynchos* orthologues were performed using the MUSCLE [64]. The alignments were translated into a codon alignment with the Perl script PAL2NAL [72]. Non-synonymous (Ka) and synonymous (Ks) substitution rates were calculated using KaKs_Calculator 2.0 [73] with default parameters.

### Syntenic relationship with the *A. platyrhynchos* genome

To assess the syntenic relationship with the *A. platyrhynchos* genome, we used minimap2 (-x asm5) [74] to align the assembled pseudomolecules corresponding to the chromosomes to the *A. platyrhynchos* chromosomes. The result was then visualized as a dot plot using D-GENIES [75] to assess the synteny relationship, as well as identifying regions exhibiting collinearity and rearrangements between the two assemblies. We then used two different methods to verify these structural variations. First, Muscovy duck and *A. platyrhynchos* genomes were aligned to identify rearrangements using SyRI [32] with default parameters. Second, we aligned trimmed 10x Genomics barcode reads to the *A. platyrhynchos* genome using BWA (v-0.7.12) [76], after which the Breakdancer (v1.4.5) [31] software was used to detect structural variations.

### Divergence and polymorphism analyses

We extracted 1:1 orthology of Muscovy-*A. platyrhynchos* orthologous genes from the OrthoFinder results. Multiple protein sequence alignments of orthologues were performed using MUSCLE [64]. The codeml program from the PAML package [67] was used to estimate the non-synonymous substitution rates (dN) and the synonymous substitution rates (dS) for Z chromosomes and autosomes separately. The orthologous genes with abnormal dS values (< 0.001) were removed as too few synonymous sites/substitutions indicate that those genes were probably misaligned. Confidence intervals (95 %) were calculated using bootstrapping (1,000).

We retrieved re-sequencing data for 4 Chinese Muscovy ducks [77] and 6 wild mallards from a previous study [78], available from the NCBI (SRP144280). The sequencing depth for each specimen was more than 10x. Raw reads were aligned to the Muscovy genome using BWA-MEM [76] with default parameters. Samtools (v-1.3.1) [79] software was used to sort the alignment bam files and the repeated reads were removed by a Picard tools MarkDuplicates (v-1.108) [80]. Polymorphic positions were called using Genome Analysis Toolkit (GATK, v-3.5) [81]. Subsequently, we used it to filter these variants with the following parameters: “QUAL > 30.0, QD > 2.0, FS < 60.0, MQ > 40.0, MQRankSum > -12.5, ReadPosRankSum > -8.0, SOR > 10.0, clusterWindowSize = 10, --clusterSize = 3”. Furthermore, the plink (v-1.9) software [82] with a parameter (--geno 0.1) was used to filter the above SNPs again to remove false positives.

Based on the above SNPs (autosomes), a phylogenetic tree was constructed using the SNPhylo (v-20,140,701) software [83]. SnpEff software (v-4.3t) [84] was used to identify variants located at protein-coding positions and whether they were synonymous or non-synonymous. Then, population fixation statistics (F_ST_), non-synonymous (πn) and synonymous (πs) nucleotide diversity was computed for each coding gene using VCFtools software (v-0.1.15) [85] with the parameter (--site-pi). To screen for inversion polymorphisms, we calculated F_ST_ values using VCFtools, with windows of 40 kb in length sliding across the genome. Linkage disequilibrium (LD) values among SNPs were inferred using LDBlockShow (-MAF 0.4 -Het 0.8) [86].

## Supplementary Information

**Additional file 1: Figure S1.** Histogram of the 21-mer depth distribution of the sequencing reads of Muscovy duck plotted in GenomeScope. The kmer with coverage of 50X has the largest peak (excluding the kmer with extremely low coverage), which was used to estimate the genome size.

**Additional file 2: Table S1.** Descriptive metrics, estimated by Supernova, of the input sequence data for the de novo genome assembly.

**Additional file 3: Table S2.** Summary statistics of ab-initio*,* homology-based and RNA-seq based gene prediction results.

**Additional file 4: Table S3.** Integrity assessment of the coding sequence of Muscovy duck (annotated 15,580 genes).

**Additional file 5: Table S4.** Summary of identified repeat elements in the Muscovy duck genome.

**Additional file 6: Figure S2.** Venn diagram of functional annotation for the 15,580 protein-coding genes predicted in the Muscovy duck genome. The numbers indicate the numbers of genes in the Muscovy duck genome identified in different databases. NR: the non-redundant protein sequences database in NCBI. NT: the nucleotide database in NCBI.

**Additional file 7: Table S5.** OrthoFinder gene counts per orthogroup.

**Additional file 8: Table S6.** 762 Muscovy duck-specific genes.

**Additional file 9: Figure S3.** GO term annotation of Muscovy duck species-specific genes.

**Additional file 10: Table S7.** Functional annotation of Muscovy duck species-specific genes.  

**Additional file 11: Table S8.** The list of 134 rapidly expanded gene families and 529 contracted gene families.

**Additional file 12: Table S9.** Functional annotation of 39 genes in Muscovy duck.

**Additional file 13: Table S10.** 104 positively selected genes (PSGs) in Muscovy duck.

**Additional file 14: Table S11.** Functional annotation of positively selected genes in the Muscovy duck genome.

**Additional file 15: Table S12.**  Genes involved in the innate immune response pathway.

**Additional file 16: Table S13.** Breakdancer software was used to verify structural variations.

**Additional file 17: Table S14.** SyRI software was used to verify inversion.

**Additional file 18: Figure S4.** Structural variations detected using SyRI software. INV refers to inversions, TRANS refers to transpositions, SNP refers to single-nucleotide polymorphism, DEL refers to deletions. The shaded area is a 95% confidence interval.

**Additional file 19: Figure S5.** Phylogenetic tree was constructed using whole genome SNP data. The red and blue are Muscovy and mallard populations, respectively.

**Additional file 20: Table S15.** The assembly accession of the seven species genome in the NCBI public database.

## Data Availability

The Muscovy duck genome sequences in this study have been deposited with links to BioProject accession number PRJNA669953 in the NCBI BioProject database (https://www.ncbi.nlm.nih.gov/bioproject/PRJNA669953). The transcriptome data for genome annotation is available with links to BioProject accession number PRJNA678766 (https://www.ncbi.nlm.nih.gov/bioproject/PRJNA678766). The Whole Genome project has been deposited at GenBank under the accession JADZGK000000000 (https://www.ncbi.nlm.nih.gov/assembly/JADZGK000000000).

## Abbreviations

BUSCO: Benchmarking Universal Single-Copy Orthologs
TEs: Transposable elements
GO: Gene Ontology
KEGG: Kyoto Encyclopedia of Genes and Genomes
MYA: Million years ago
Ka: Nonsynonymous substitutions
Ks: Synonymous substitutions
SNP: Single nucleotide polymorphism
dN: Non-synonymous substitution rates
dS: Synonymous substitution rates
Ne: Effective population size
πn: Non-synonymous nucleotide diversity
πs: Synonymous nucleotide diversity
NR: Non-redundant protein
NT: Nucleotide Sequence
BWA: Burrows-Wheeler aligner
GATK: Genome Analysis Toolkit
F_ST_: Fixation statistics
