# Supplementary material for: A chromosome-level genome assembly of Cairina moschata and comparative genomic analyses
Source: BMC Genomics. 2021 Jul 30;22:581. doi: 10.1186/s12864-021-07897-4 (PMC8325232; doi:10.1186/s12864-021-07897-4)
Supplement: Supplementary file 1 — Additional file 1: Figure S1. Histogram of the 21-mer depth distribution of the sequencing reads of Muscovy duck plotted in GenomeScope. The kmer with coverage of 50X has the largest peak (excluding the kmer with extremely low coverage), which was used to estimate the genome size. [file 12864_2021_7897_MOESM1_ESM.pdf]

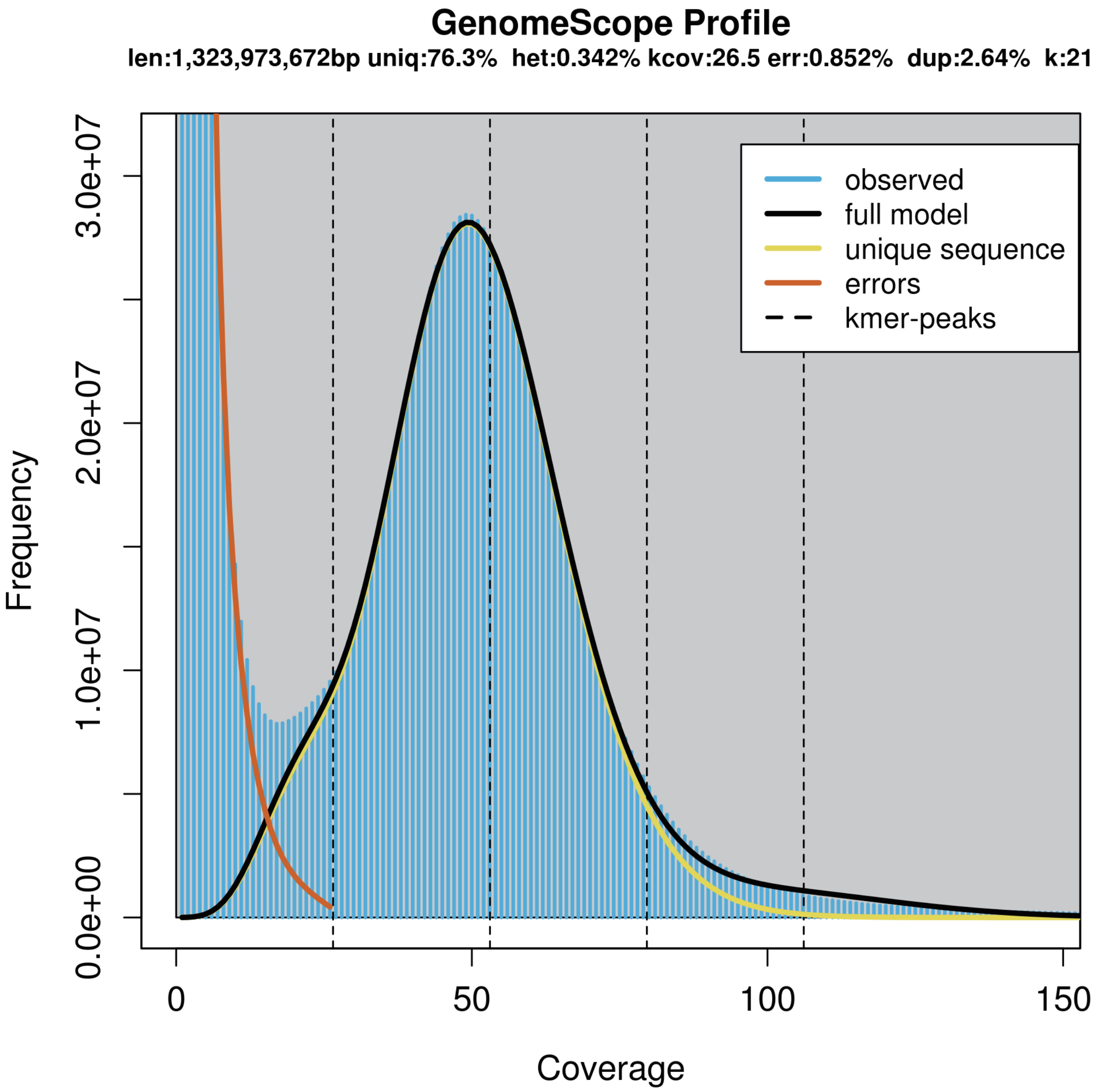

Fig. S1 Histogram of the 21-mer depth distribution of the sequencing reads of Muscovy duck plotted in GenomeScope. The kmer with coverage of 50X has the largest peak (excluding the kmer with extremely low coverage), which was used to estimate the genome size.
