# Supplementary material for: A chromosome-level genome assembly of Cairina moschata and comparative genomic analyses
Source: BMC Genomics. 2021 Jul 30;22:581. doi: 10.1186/s12864-021-07897-4 (PMC8325232; doi:10.1186/s12864-021-07897-4)
Supplement: Supplementary file 2 — Additional file 2: Table S1. Descriptive metrics, estimated by Supernova, of the input sequence data for the de novo genome assembly. [file 12864_2021_7897_MOESM2_ESM.docx]

Table S1. Descriptive metrics, estimated by Supernova, of the input sequence data for the de novo genome assembly

| **Metric** | **Value** |
| --- | --- |
| Number of paired reads used | 858.04 Mb |
| Mean read length after trimming | 138.50 bp |
| Median insert size | 384.00 bp |
| Weighted mean DNA molecule size | 16.65 kb |
| Raw coverage | 85.49 X |
| Effective read coverage | 54.82 X |
| Mean distance between heterozygous SNPs | 419.00 bp |
