# Supplementary material for: A chromosome-level genome assembly of Cairina moschata and comparative genomic analyses
Source: BMC Genomics. 2021 Jul 30;22:581. doi: 10.1186/s12864-021-07897-4 (PMC8325232; doi:10.1186/s12864-021-07897-4)
Supplement: Supplementary file 3 — Additional file 3: Table S2. Summary statistics of ab-initio, homology-based and RNA-seq based gene prediction results. [file 12864_2021_7897_MOESM3_ESM.docx]

Table S2. Summary statistics of ab initio*,* homology-based and RNA-seq based gene prediction results

| **Evidence Type** | **Programs** | **Element** | **Total count** | **Exon/Gene** | **Total length (bp)** | **Mean length (bp)** |
| --- | --- | --- | --- | --- | --- | --- |
| ab-initio | Augustus | gene | 39,873 | 7.19 | 780,466,264 | 19573.8 |
|  |  | exon | 286,488 |  | 54,829,721 | 191.39 |
|  | GeneMark-ES | gene | 77,341 | 12.49 | 339,301,869 | 4387.09 |
|  |  | exon | 966,114 |  | 83,904,997 | 86.85 |
| homology | Exonerate | gene | 560,716 | 4.63 | 11,755,113,245 | 20964.47 |
|  |  | exon | 2,593,767 |  | 610,890,792 | 235.52 |
| RNA-seq |  | gene | 49,377 | 9.32 | 1,640,301,863 | 33219.96 |
|  |  |  |  |  |  |  |
|  | Hisat2+Stringtie | exon | 460,275 |  | 155,349,976 | 337.52 |
|  |  | gene | 91,875 | 9.55 | 2,693,994,634 | 29322.39 |
|  |  |  |  |  |  |  |
|  | PASA+TransDecoder | exon | 877,727 |  | 345,181,379 | 393.27 |
| ab-initio+Homology+RNAseq(EVM filter) | | gene | 15,581 | 10.18 | 335,977,078 | 21563.26 |
|  |  | exon | 158,585 |  | 25,294,638 | 159.5 |
