# Supplementary material for: A chromosome-level genome assembly of Cairina moschata and comparative genomic analyses
Source: BMC Genomics. 2021 Jul 30;22:581. doi: 10.1186/s12864-021-07897-4 (PMC8325232; doi:10.1186/s12864-021-07897-4)
Supplement: Supplementary file 4 — Additional file 4: Table S3. Integrity assessment of the coding sequence of Muscovy duck (annotated 15,580 genes). [file 12864_2021_7897_MOESM4_ESM.docx]

Table S3. Integrity assessment of the coding sequence of Muscovy duck (annotated 15,580 genes)

| **Category** | **Number of BUSCOs** |
| --- | --- |
| Complete BUSCOs (C) | 7567 |
| Complete and single-copy BUSCOs (S) | 7461 |
| Complete and duplicated BUSCOs (D) | 106 |
| Fragmented BUSCOs (F) | 289 |
| Missing BUSCOs (M) | 482 |
| Total BUSCO groups searched | 8338 |
| C:90.8%[S:89.5%,D:1.3%],F:3.5%,M:5.7%,n:8338 | |
