# Supplementary material for: A chromosome-level genome assembly of Cairina moschata and comparative genomic analyses
Source: BMC Genomics. 2021 Jul 30;22:581. doi: 10.1186/s12864-021-07897-4 (PMC8325232; doi:10.1186/s12864-021-07897-4)
Supplement: Supplementary file 5 — Additional file 5: Table S4. Summary of identified repeat elements in the Muscovy duck genome. [file 12864_2021_7897_MOESM5_ESM.docx]

Table S4. Summary of identified repeat elements in the Muscovy duck genome

| **Repeat Element** | **No. Element** | **Length. Element (bp)** | **%** |
| --- | --- | --- | --- |
| SINEs | 2,740 | 279,651 | 0.02% |
| LINEs | 124,478 | 53,824,424 | 4.81% |
| LTR elements | 32,626 | 17,022,567 | 1.52% |
| Penelope | 803 | 91,334 | 0.01% |
| DNA transposons | 10,998 | 1,162,851 | 0.10% |
| Small RNA | 716 | 84,646 | 0.01% |
| Simple repeat | 506,289 | 18,975,451 | 1.70% |
| Low complexity | 77,630 | 3,751,875 | 0.34% |
| Unclassified | 20,686 | 7,613,487 | 0.68% |
| Total | 776,966 | 102,806,286 | 9.19% |
