# Supplementary material for: A chromosome-level genome assembly of Cairina moschata and comparative genomic analyses
Source: BMC Genomics. 2021 Jul 30;22:581. doi: 10.1186/s12864-021-07897-4 (PMC8325232; doi:10.1186/s12864-021-07897-4)
Supplement: Supplementary file 6 — Additional file 6: Figure S2. Venn diagram of functional annotation for the 15,580 protein-coding genes predicted in the Muscovy duck genome. The numbers indicate the numbers of genes in the Muscovy duck genome identified in different databases. NR: the non-redundant protein sequences database in NCBI. NT: the nucleotide database in NCBI. [file 12864_2021_7897_MOESM6_ESM.pdf]

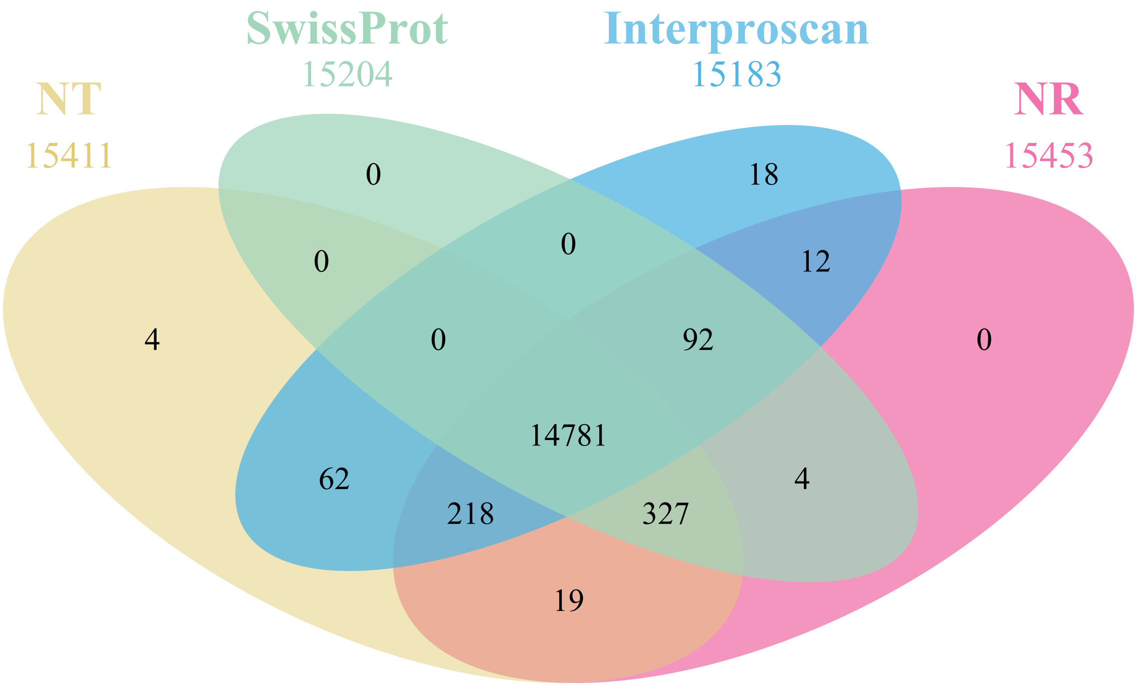

Fig. S2 Venn diagram of functional annotation for the 15,580 protein-coding genes predicted in the Muscovy duck genome. The numbers indicate the numbers of genes in the Muscovy duck genome identified in different databases. NR: the non-redundant protein sequences database in NCBI. NT: the nucleotide database in NCBI.
