# Supplementary figures and images for: A chromosome-level genome assembly of Cairina moschata and comparative genomic analyses
Source: BMC Genomics. 2021 Jul 30;22:581. doi: 10.1186/s12864-021-07897-4 (PMC8325232; doi:10.1186/s12864-021-07897-4)

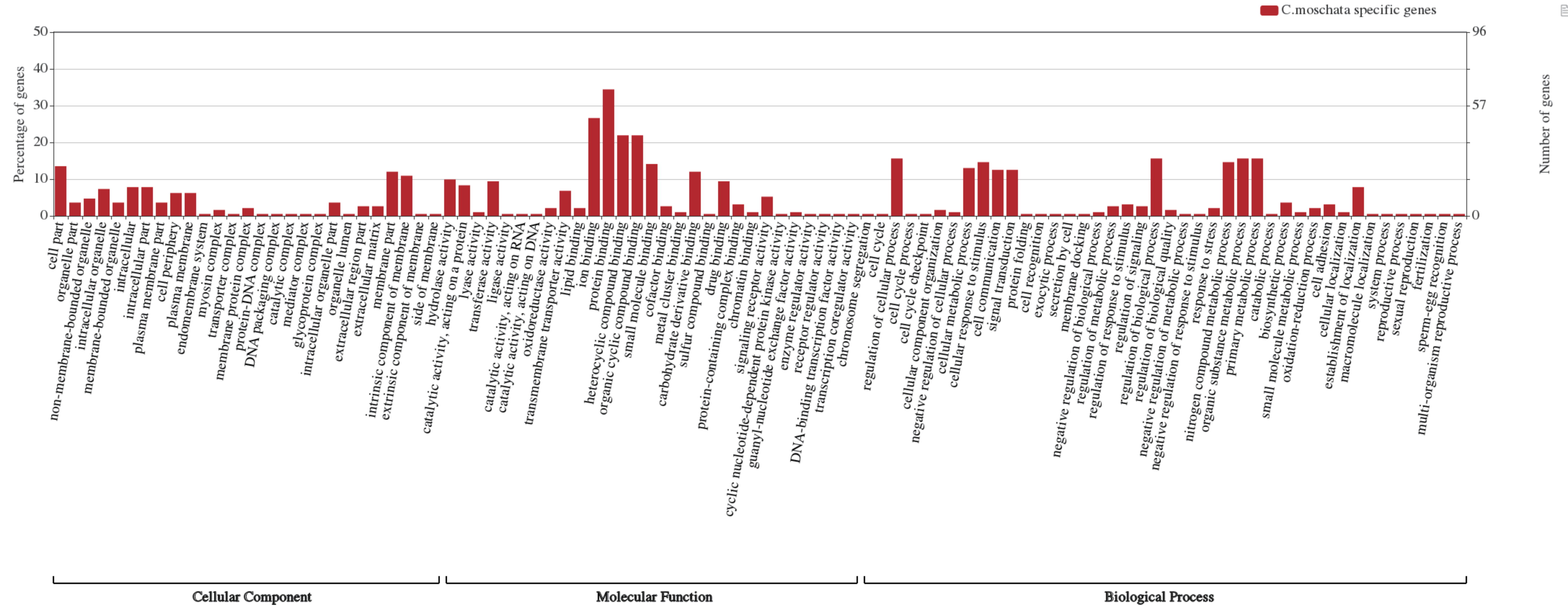

**Fig. S3** GO term annotation of Muscovy duck species-specific genes.

Supplement: Supplementary file 9 — Additional file 9: Figure S3. GO term annotation of Muscovy duck species-specific genes. [file 12864_2021_7897_MOESM9_ESM.pdf]
