# Supplementary material for: A chromosome-level genome assembly of Cairina moschata and comparative genomic analyses
Source: BMC Genomics. 2021 Jul 30;22:581. doi: 10.1186/s12864-021-07897-4 (PMC8325232; doi:10.1186/s12864-021-07897-4)
Supplement: Supplementary file 10 — Additional file 10: Table S7. Functional annotation of Muscovy duck species-specific genes. [file 12864_2021_7897_MOESM10_ESM.docx]

Table S7. Functional annotation of Muscovy duck species-specific genes

| **KEGG terms** | **Genes number** | **Background genes** | **P Value** |
| --- | --- | --- | --- |
| Calcium signaling pathway | 18 | 175 | 1.77E-07 |
| Vascular smooth muscle contraction | 14 | 113 | 5.47E-07 |
| Gap junction | 12 | 81 | 6.46E-07 |
| Focal adhesion | 15 | 188 | 3.13E-05 |
| Adherens junction | 9 | 68 | 3.66E-05 |
| Cell adhesion molecules (CAMs) | 11 | 108 | 4.75E-05 |
| AGE-RAGE signaling pathway in diabetic complications | 9 | 95 | 0.000376438 |
| Adrenergic signaling in cardiomyocytes | 10 | 118 | 0.000413323 |
| ErbB signaling pathway | 8 | 76 | 0.000414574 |
| Apelin signaling pathway | 10 | 119 | 0.000439729 |
| Purine metabolism | 10 | 120 | 0.00046752 |
| Phosphatidylinositol signaling system | 8 | 89 | 0.001082039 |
| Wnt signaling pathway | 10 | 144 | 0.001718261 |
| MAPK signaling pathway | 14 | 253 | 0.001821764 |
| ECM-receptor interaction | 7 | 84 | 0.003255262 |
| GnRH signaling pathway | 7 | 87 | 0.003900151 |
| Metabolic pathways | 41 | 1228 | 0.005162941 |
| Melanogenesis | 7 | 93 | 0.00547555 |
| Regulation of actin cytoskeleton | 10 | 176 | 0.006595354 |
| Cardiac muscle contraction | 5 | 63 | 0.014740932 |
| Oocyte meiosis | 6 | 94 | 0.019860184 |
| Fructose and mannose metabolism | 3 | 29 | 0.029914436 |
| C-type lectin receptor signaling pathway | 5 | 85 | 0.042796743 |
| Glycosphingolipid biosynthesis - ganglio series | 2 | 14 | 0.04435115 |
