# Supplementary material for: A chromosome-level genome assembly of Cairina moschata and comparative genomic analyses
Source: BMC Genomics. 2021 Jul 30;22:581. doi: 10.1186/s12864-021-07897-4 (PMC8325232; doi:10.1186/s12864-021-07897-4)
Supplement: Supplementary file 14 — Additional file 14: Table S11. Functional annotation of positively selected genes in the Muscovy duck genome. [file 12864_2021_7897_MOESM14_ESM.docx]

Table S11. Functional annotation of positively selected genes in the Muscovy duck genome

| **KEGG terms** | **Genes number** | **Background genes** | **P Value** |
| --- | --- | --- | --- |
| Toll-like receptor signaling pathway | 4 | 78 | 0.00046 |
| Cytokine-cytokine receptor interaction | 5 | 176 | 0.001195 |
| Necroptosis | 4 | 116 | 0.001903 |
| Influenza A | 4 | 119 | 0.002082 |
| Herpes simplex virus 1 infection | 4 | 124 | 0.002407 |
| NOD-like receptor signaling pathway | 3 | 110 | 0.013342 |
| Protein processing in endoplasmic reticulum | 3 | 139 | 0.024329 |
| PPAR signaling pathway | 2 | 62 | 0.031873 |
| p53 signaling pathway | 2 | 62 | 0.031873 |
