# Supplementary material for: A chromosome-level genome assembly of Cairina moschata and comparative genomic analyses
Source: BMC Genomics. 2021 Jul 30;22:581. doi: 10.1186/s12864-021-07897-4 (PMC8325232; doi:10.1186/s12864-021-07897-4)
Supplement: Supplementary file 16 — Additional file 16: Table S13. Breakdancer software was used to verify structural variations. [file 12864_2021_7897_MOESM16_ESM.docx]

Table S13. Breakdancer software was used to verify structural variations

| **Mallard Chr** | **INV start** | **INV end** | **SV size** | **Read Support** | **score** |
| --- | --- | --- | --- | --- | --- |
| Chr18 | 5,817,446 | 5,822,659 | 5,213 | 110 | 32 |
|  | 5,828,479 | 5,835,898 | 7,419 | 172 | 39 |
| Chr25 | 3,753,045 | 3,760,923 | 7,878 | 230 | 99 |
|  | 3,791,192 | 3,792,705 | 1,513 | 137 | 43 |
|  | 3,917,051 | 3,920,023 | 2,972 | 67 | 42 |
|  | 3,429,804 | 5,363,694 | 1,933,890 | 82 | 37 |
| ChrZ | 42,547,111 | 42,550,579 | 3,468 | 87 | 33 |
|  | 44,251,992 | 44,253,139 | 1,147 | 31 | 54 |
