# Supplementary material for: A chromosome-level genome assembly of Cairina moschata and comparative genomic analyses
Source: BMC Genomics. 2021 Jul 30;22:581. doi: 10.1186/s12864-021-07897-4 (PMC8325232; doi:10.1186/s12864-021-07897-4)
Supplement: Supplementary file 17 — Additional file 17: Table S14. SyRI software was used to verify inversion. [file 12864_2021_7897_MOESM17_ESM.docx]

Table S14. SyRI software was used to verify inversion

| **Muscovy Chr** | **INV start** | **INV end** | **Mallard Chr** | **INV start** | **INV end** | |
| --- | --- | --- | --- | --- | --- | --- |
| Chr18 | 6,493,112 | 8,892,652 | Chr18 | 5,443,519 | | 7,886,436 |
| Chr25 | 3,453,094 | 5,598,788 | Chr25 | 3,441,398 | | 5,367,442 |
| ChrZ | 41,334,740 | 44,694,224 | ChrZ | 39,433,445 | | 42,586,338 |
