# Supplementary material for: A chromosome-level genome assembly of Cairina moschata and comparative genomic analyses
Source: BMC Genomics. 2021 Jul 30;22:581. doi: 10.1186/s12864-021-07897-4 (PMC8325232; doi:10.1186/s12864-021-07897-4)
Supplement: Supplementary file 18 — Additional file 18: Figure S4. Structural variations detected using SyRI software. INV refers to inversions, TRANS refers to transpositions, SNP refers to single-nucleotide polymorphism, DEL refers to deletions. The shaded area is a 95% confidence interval. [file 12864_2021_7897_MOESM18_ESM.pdf]

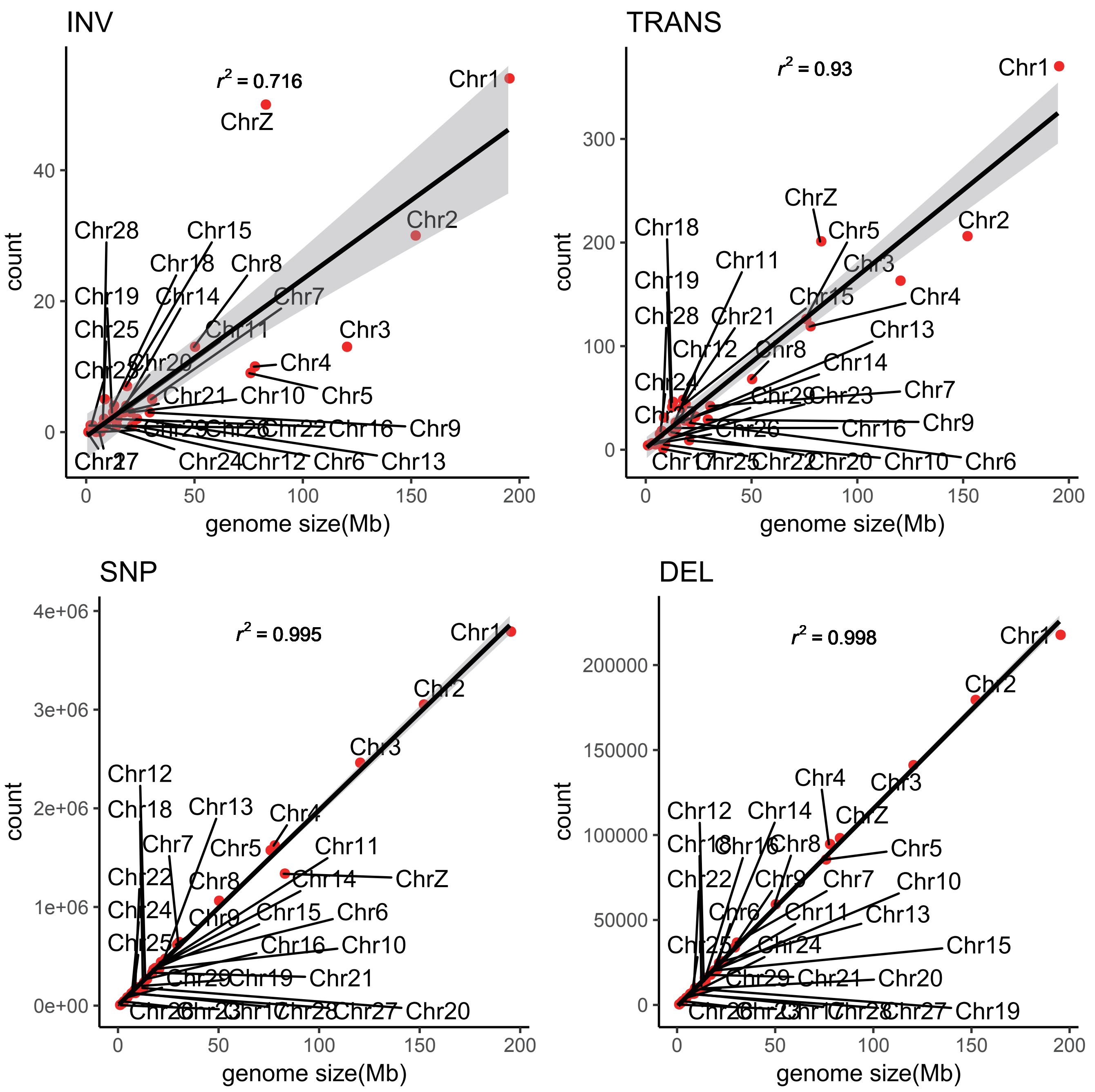

Fig. S4 Structural variations detected using SyRI software. INV refers to inversions, TRANS refers to transpositions, SNP refers to single-nucleotide polymorphism, DEL refers to deletions. The shaded area is a 95% confidence interval.
