# Supplementary material for: A chromosome-level genome assembly of Cairina moschata and comparative genomic analyses
Source: BMC Genomics. 2021 Jul 30;22:581. doi: 10.1186/s12864-021-07897-4 (PMC8325232; doi:10.1186/s12864-021-07897-4)
Supplement: Supplementary file 19 — Additional file 19: Figure S5. Phylogenetic tree was constructed using whole genome SNP data. The red and blue are Muscovy and mallard populations, respectively. [file 12864_2021_7897_MOESM19_ESM.pdf]

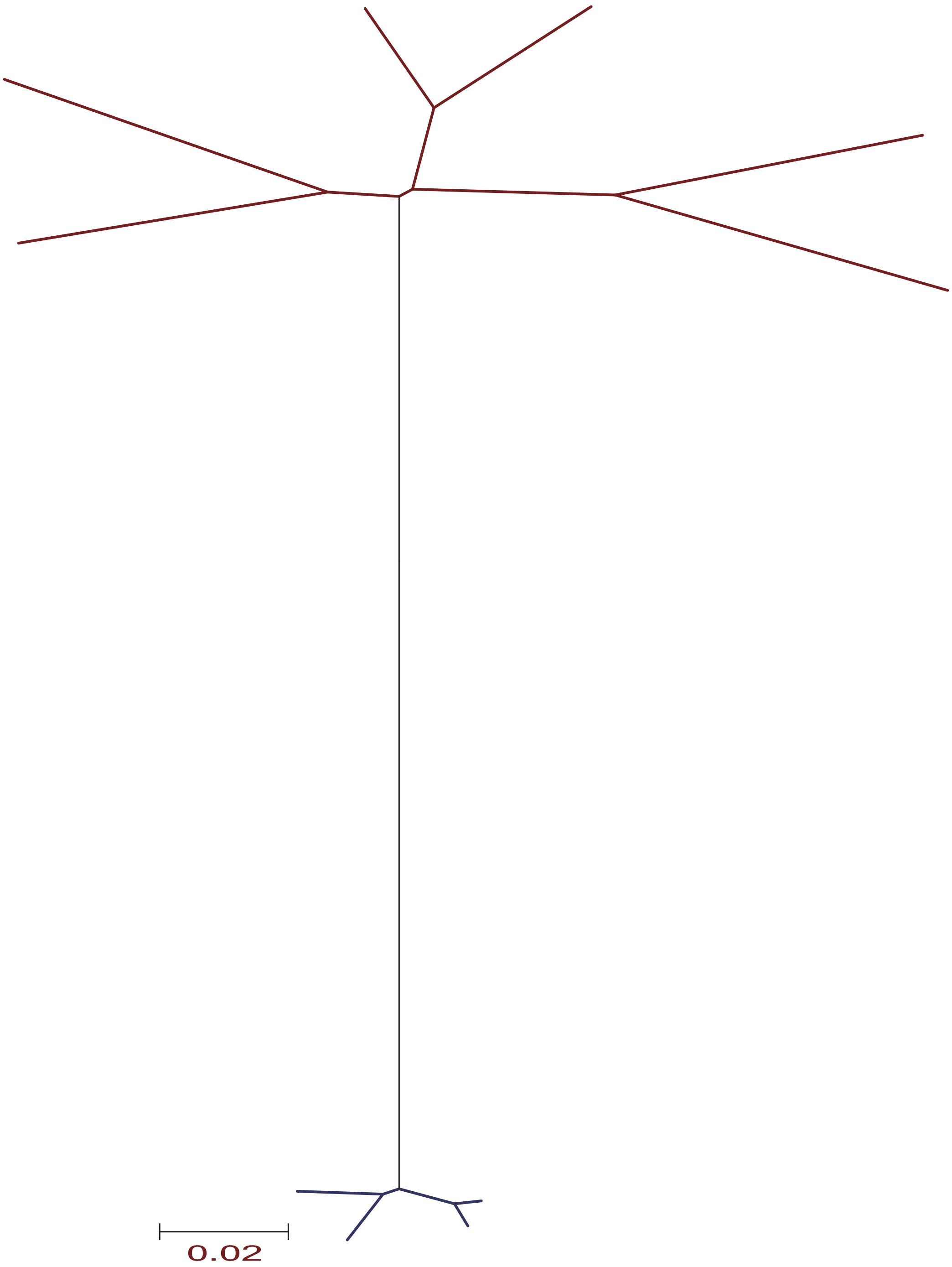

Fig. S5 Phylogenetic tree was constructed using whole genome SNP data. The red and blue are Muscovy and mallard populations, respectively.
