# Supplementary material for: A chromosome-level genome assembly of Cairina moschata and comparative genomic analyses
Source: BMC Genomics. 2021 Jul 30;22:581. doi: 10.1186/s12864-021-07897-4 (PMC8325232; doi:10.1186/s12864-021-07897-4)
Supplement: Supplementary file 20 — Additional file 20: Table S15. The assembly accession of the seven species genome in the NCBI public database. [file 12864_2021_7897_MOESM20_ESM.docx]

Table S15. The assembly accession of the seven species genome in the NCBI public database

| **Species** | **Assembly Accession (NCBI)** |
| --- | --- |
| *Taeniopygia guttata* | GCA_008822105.2 |
| *Anser cygnoides* | GCA_000971095.1 |
| *Anas platyrhynchos* | GCA_003850225.1 |
| *Meleagris gallopavo* | GCA_000146605.4 |
| *Gallus gallus* | GCA_000002315.5 |
| *Mus musculus* | GCA_000001635.9 |
| *Homo sapiens* | GCA_000001405.28 |
